# Fault-Tolerant Cooperative Positioning for UAV Swarms in Degraded Environments: A Multi-Objective Deep Reinforcement Learning Approach

**DOI:** 10.3390/s26123747

**Published:** 2026-06-12

**Authors:** Peiru Yang, Jiayong Li, Xiaoyang Lan, Bao Pang

**Affiliations:** School of Airspace Science and Engineering, Shandong University, Weihai 264200, China; 202300800202@mail.sdu.edu.cn (P.Y.); 202300800272@mail.sdu.edu.cn (J.L.); 202300800293@mail.sdu.edu.cn (X.L.)

**Keywords:** UAV swarm, cooperative localization, deep reinforcement learning (DRL), extended Kalman filter (EKF), fault tolerance, multi-objective optimization, error contagion isolation

## Abstract

**Highlights:**

**What are the main findings?**

- Proposes a novel MADRL-CEKF framework designed for highly resilient micro UAV swarm cooperative localization in severe GNSS-denied and obstacle-dense environments.
- Introduces a link-level dynamic soft isolation mechanism that independently evaluates ranging links, effectively severing the contagion paths of cascading cooperative errors.

**What are the implications of the main findings?**

- Integrates an adaptive Markov smoothing constraint to bridge discrete high-level AI decisions with low-level filtering, eliminating high-frequency control jitter.
- Develops a resource-aware multi-objective reward architecture, cutting processing delay and energy footprint by over 40% to strictly meet the 50 ms real-time limit.

**Abstract:**

When operating in complex and obstacle-dense environments, micro UAV swarms often face severe cooperative positioning failures due to transient non-line-of-sight (NLOS) interference and cascaded inertial sensor drift. To address this, this work proposes a fault-tolerant positioning framework integrating multi-agent deep reinforcement learning with cooperative extended Kalman filtering (MADRL-CEKF). The system incorporates a link-level dynamic soft isolation mechanism that dynamically adjusts observation covariance to effectively sever paths of cooperative error contagion. An adaptive Markov smoothing constraint is mathematically embedded to mitigate high-frequency control jitter typical of AI-driven policies. Crucially, the framework implements a resource-aware multi-objective reward architecture tailored for micro UAVs. Evaluated through high-fidelity simulations and offline physical datasets, the proposed framework achieves a 96.01% reduction in average tracking error (RMSE) under extreme multi-node cascaded failures, completely preventing system divergence. Furthermore, through autonomous multi-objective trade-offs, the system reduces processing delay by 44% (to 25.1 ms) and computational energy consumption by 41% with only a marginal accuracy compromise of 0.16 m, strictly keeping the execution time within the 50 ms real-time threshold. The MADRL-CEKF framework effectively bridges the gap between sophisticated AI decision-making and strict engineering constraints, providing a highly robust and resource-efficient navigation paradigm for swarm robotics.

## 1. Introduction

In recent years, micro unmanned aerial vehicle (UAV) swarm technology has been steadily advancing. Its autonomous operational capability in Global Navigation Satellite System (GNSS)-denied environments has become a research hotspot in robotics [1,2]. Extreme scenarios include post-disaster rescue in earthquake-collapsed structures, subterranean exploration of deep mining shafts, and complex industrial inspections involving nuclear facilities [3,4]. In such environments, UWB-based cooperative positioning systems typically achieve 0.5–1 m accuracy in open indoor spaces [5,6]. However, dense concrete occlusions often increase positioning errors to 5–10 m, and may even lead to complete failure [7,8]. Therefore, deploying resilient UAV swarms is of great importance, effectively protecting personnel from secondary hazards and accelerating time-critical damage assessment. Swarm positioning faces unique challenges absent in single-UAV systems: the high interdependence among nodes means that a single erroneous measurement can rapidly propagate through the communication network, potentially causing catastrophic system-level failure. Ensuring the positioning reliability of each UAV node is therefore a prerequisite for executing coordinated swarm tasks.

Currently, the mainstream approach for indoor multi-agent positioning is the tightly coupled cooperative extended Kalman filter (CEKF) based on UWB and IMU data [9,10]. To handle measurement anomalies, traditional robust methods rely on factor graph optimization (FGO) [11,12], particle filters (PFs) [13], and outlier rejection algorithms based on M-estimators (e.g., Huber kernel) and RANSAC [14]. However, real-world harsh environments are typically filled with dense physical occlusions and electromagnetic interference, leading to spatially correlated, highly nonlinear transient non-line-of-sight (NLOS) errors or permanent hardware drift. Under such extreme conditions, conventional robust methods reveal fatal limitations. Standard CEKF is highly dependent on pre-calibrated, fixed covariance matrices and is completely defenseless against sudden anomalies. Meanwhile, methods like Huber-based robust filtering and RANSAC rely heavily on rigid, heuristic mathematical thresholds. While they can mitigate occasional outliers, they fail to adapt to sustained, dynamically changing nonlinear disturbances, easily misleading the system into blindly absorbing corrupted ranging measurements. Consequently, local measurement errors quickly propagate and amplify through the swarm’s communication topology, eventually triggering a catastrophic cascading failure of the global positioning system.

To overcome the rigidness of traditional filters, the research community has recently attempted to incorporate artificial intelligence into adaptive filter parameter optimization [15,16]. Several pioneering works have utilized graph neural networks (GNNs) [17,18] and multi-agent deep reinforcement learning (MADRL) [19,20] to assess node trustworthiness in cooperative positioning. Nonetheless, a critical review of the current literature exposes three major gaps preventing their deployment in extreme swarm scenarios. First, the granularity of existing isolation mechanisms is excessively coarse. Most GNN-based fault tolerance schemes conduct node-level trust assessments. If a node is deemed anomalous, the system enforces a hard isolation, discarding all of its spatial connections. This approach is highly inefficient, as it blindly abandons the healthy ranging links of a partially compromised UAV. Second, standard DRL-based methods lack physical control continuity. Purely algorithmic derivations tend to treat filtering merely as a numerical optimization problem, overlooking the frequency oscillation characteristics of real physical systems. Abrupt and erratic alterations in the neural network’s weight outputs invariably introduce severe high-frequency jitter at the control level, destabilizing flight controllers. Third, current AI-enhanced localization techniques suffer from severe resource blindness. Micro UAV nodes are characterized by strictly limited computing power and battery capacities [21,22]. Current state-of-the-art approaches excessively pursue millimeter-level accuracy but completely ignore the computational delay and energy footprint. For instance, heavy RL approaches based on transformers involve massive tensor operations exceeding 50 ms [23,24], rendering them totally impractical for high-frequency real-time execution.

To systematically address these critical limitations, this paper proposes a resilient cooperative positioning framework for UAV swarms in denied environments by deeply fusing MADRL and CEKF. Rather than executing crude node-level disconnection, the proposed approach implements a link-level dynamic soft isolation. Innovation residuals are calculated by each UAV node based on inertial propagations and UWB measurements. The MADRL policy network then independently evaluates the degradation of each specific communication path, outputting adaptive trust weights to mathematically inflate the corresponding observation covariance. Furthermore, to bridge the gap between discrete AI decisions and continuous physical states, an adaptive Markov smoothing constraint is embedded as a temporal low-pass filter, completely eliminating erratic control jitter. Finally, a resource-aware multi-objective optimization architecture is explicitly designed into the training phase, forcing the agent to autonomously balance localization accuracy, execution latency, and energy cost.

To explicitly clarify the distinct boundaries and advantages of the proposed framework over existing state-of-the-art literature, a comprehensive comparison is summarized in Table 1. While conventional robust EKF methods rely on heuristic and rigid adaptive covariance tuning, they lack dynamic adaptability to highly nonlinear disturbances. Recent GNN-based methods typically perform node-level trust evaluation, which fails to isolate specific faulty links without completely discarding the node. Furthermore, although existing DRL-based cooperative localization methods demonstrate strong nonlinear fitting capabilities, they rarely address the high-frequency control jitter caused by algorithmic outputs, nor do they consider the stringent computational and energy constraints of micro UAVs. In contrast, the proposed MADRL-CEKF framework uniquely integrates link-level soft isolation, an adaptive Markov smoothing constraint to eliminate decision jitter, and a multi-objective reward mechanism that explicitly ensures energy and computational awareness.

The primary contributions of this work are summarized as follows:

1. A Link-Level Dynamic Soft Isolation Mechanism Based on MADRL. Unlike traditional heuristic or node-level isolation methods, the proposed scheme independently evaluates the trust of each UWB ranging link. This enables the selective isolation of faulty links while preserving healthy ones, substantially enhancing network robustness.
2. An Adaptive Markov Smoothing Constraint for Decision Continuity. To bridge discrete high-level AI decisions with continuous low-level actuator constraints, we introduce a dynamic smoothing mechanism. It adjusts the smoothing constant based on the swarm’s real-time status, mitigating sudden fluctuations in decision weights typical of conventional fixed-parameter filters.
3. A Resource-Aware Multi-Objective Optimization Architecture. Addressing the strict computational and power limits of micro UAVs, this architecture implements a multi-dimensional reward structure that considers accuracy, processing delay, and energy costs. The agent autonomously balances these objectives, ensuring that single-run execution times remain safely within the 50 ms real-time threshold.

Because of the extremely high danger associated with UAV crashes and because controlled experimentation in an extreme scenario is highly challenging (such as in a highly obstructive environment such as ruin structures), the proposed framework was mainly validated through simulations. The environment was set up using actual sensor readings and hardware parameters that allowed for validation of the framework under extremely hazardous circumstances. Seven different simulations were carried out to prove the framework’s efficacy:

1. Baseline performance comparison in ideal environments;
2. Real-world robustness validation on the public MILUV dataset;
3. Swarm scalability and error contagion analysis with 2/4/6/8 UAV nodes;
4. XAI-based dynamic trust allocation and decision interpretability analysis;
5. System robustness testing under cascaded multi-node IMU anomalies;
6. Dual ablation experiments: quantifying contributions of weight smoothing and link isolation mechanisms;
7. Multi-objective reward sub-ablation and engineering performance trade-off analysis.

Multiple objective reward sub-ablation analysis: performance assessment with respect to computation time and energy cost.

Performance parameters include global RMSE, trajectory CDF, weight variance, computation delay per cycle, and energy cost. The key comparisons for testing the performance are standard CEKF and its traditional alternatives like inertial navigation alone and weighted CEKF.

The remainder of this paper is organized as follows. Section 2 details the proposed MADRL-CEKF methodological framework, including the dynamic soft isolation mechanism, adaptive Markov smoothing, and the multi-objective MDP modeling. Section 3 presents comprehensive experimental results, covering baseline comparisons, real-world dataset validation, swarm scalability, XAI interpretability, and cascaded fault tolerance. Section 4 provides an in-depth discussion of the engineering trade-offs, algorithmic interpretability, theoretical asymptotic complexity, and current limitations. Finally, Section 5 concludes the paper.

## 2. Materials and Methods

This section presents the design process of the proposed resilient cooperative localization scheme utilizing the principles of multi-agent deep reinforcement learning (MADRL). Initially, a dual-loop cooperative extended Kalman filter (CEKF) model for the UAV swarm is established, and the mathematical mechanism by which a static covariance matrix induces cascading failures is analyzed. Subsequently, the MDP model of MADRL is formally defined, taking into account both the network architecture and multi-objective reward functions. The dynamic soft isolation algorithm based on Markov smoothing constraints is derived.

### 2.1. CEKF Model and Vulnerability Analysis

**Figure 1:**
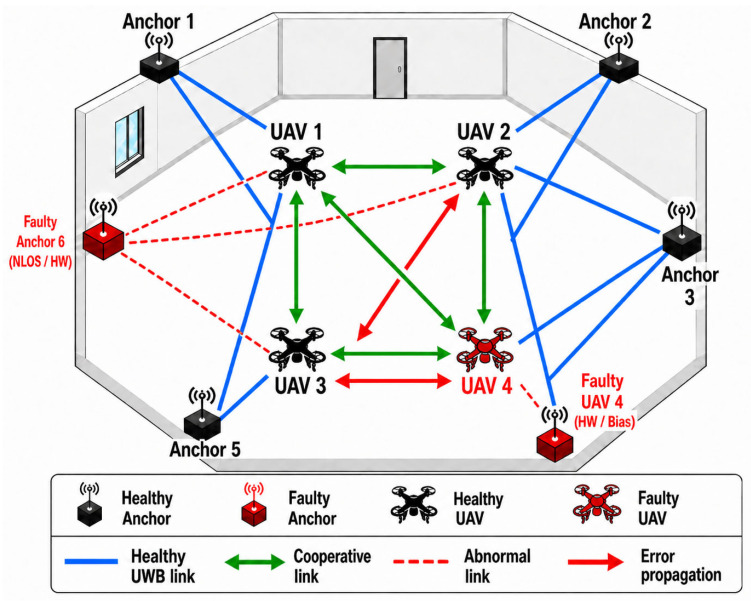
System architecture and cooperative topology of the micro UAV network.

As shown in Figure 1, the indoor cooperative system consisting of N micro UAVs and M fixed UWB anchors is based on the frequent kinematic prediction of the motion trajectory using IMU and the rare ranging correction by UWB sensors.

#### 2.1.1. State Prediction via IMU

The figure illustrates the spatial deployment of fixed UWB anchors and the micro UAV swarm in an indoor environment. It visually highlights a critical system vulnerability-error contagion: ranging anomalies originating from a faulty UWB anchor (Anchor 6) or a drifting drone (Faulty UAV 4) rapidly propagate through the network via corrupted measurement links (dashed red lines) and cascade to healthy neighboring nodes (solid red arrows), eventually degrading the global cooperative localization performance if observation covariances remain static.

For any UAV i∈{1,2,…,N}, its discrete-time state vector at time step k is defined as $X_{i}(k)=[p_{i}^{T},v_{i}^{T}{]}^{T}\in R^{6}$, where $p_{i}$ and $v_{i}$ denote the 3D position and velocity, respectively. According to inertial navigation kinematics based on the classical constant-acceleration (CA) model [25], the state transition equation is expressed as:

(1) $$X_{i}(k|k-1)=FX_{i}(k-1|k-1)+Bu_{i}(k)+w_{i}(k)$$

where $F\in R^{6\times 6}$ is the state transition matrix describing the temporal evolution of position and velocity, B is the control input matrix mapping the IMU-measured local accelerations to velocity increments in the global frame, $u_{i}(k)$ is the 3D acceleration from the IMU, and $w_{i}(k)∼N(0,Q)$ denotes process noise with covariance Q. The predicted error covariance matrix is concurrently updated as:

(2) $$P_{i}(k|k-1)=FP_{i}(k-1|k-1)F^{T}+Q$$

#### 2.1.2. Cooperative Ranging Observation

When UAV i receives a UWB signal from a topological neighbor j (fixed anchor or other UAV), the ranging observation is modeled as:

(3) $$Z_{i,j}(k)=h(X_{i}(k),X_{j}(k))+v_{i,j}(k)=\;∥p_{i}(k)-p_{j}(k){∥}_{2}+b_{NLOS}+\epsilon (k)$$

where $∥\cdot {∥}_{2}$ denotes the Euclidean distance in three-dimensional space, $\epsilon (k)∼N(0,R_{c})$ represents the constant observation noise, and $b_{NLOS}$ is the positive NLOS bias induced by physical obstacles [26] such as walls or metallic structures.

#### 2.1.3. Vulnerability of Standard Update

In conventional CEKF [2,3] updates, the innovation residual and Kalman gain $K_{i}(k)$ are computed as:

(4) $$y_{i,j}(k)=Z_{i,j}(k)-h(X_{i}(k|k-1),X_{j}(k))$$

(5) $$K_{i}(k)=P_{i}(k|k-1)H^{T}{\left(HP_{i}(k|k-1)H^{T}+R\right)}^{-1}$$

(6) $$X_{i}(k|k)=X_{i}(k|k-1)+K_{i}(k)y_{i,j}(k)$$

where H is the Jacobian of h(⋅) evaluated at the prior estimate, and $R_{i,j}$ is the observation noise covariance for the link between UAV i and UAV j. In conventional implementations, $R_{i,j}$ is conservatively set as a static scalar matrix. Under extreme NLOS interference ($b_{NLOS}\gg 0$) or severe kinematic drift of node j due to hardware failure, the rigid $R_{i,j}$ cannot penalize anomalous innovations $y_{i,j}(k)$. This leads to blind trust in $K_{i}(k)$ toward corrupted measurements, causing the state estimate of UAV i to diverge.

For instance, consider a typical UWB system with a static $R_{i,j}=0.1{\;m}^{2}$ (corresponding to a standard deviation of 0.3 m). If a hardware fault induces a 10 m ranging error, the innovation residual $y_{i,j}$ becomes 10 m. Substituting into the Kalman gain formula, the filter assigns nearly 99% weight to this erroneous measurement, almost completely disregarding the prior state estimate. This explains mathematically how the standard CEKF algorithm may experience catastrophic failures under severe conditions.

Under the cooperative network model, this type of failure would have been even further exacerbated by propagation. Information received from the malfunctioning UAV node spreads error contagion to healthy nodes. To address this inherent vulnerability, this work proposes a MADRL-based dynamic trust evaluation mechanism that adaptively adjusts $R_{i,j}$ for each link in real-time, thereby preventing error propagation and cascading failures.

### 2.2. Deep Reinforcement Learning Network Architecture and Decision Modeling

The collapse of conventional cooperative positioning algorithms originates from the rigidity of the observation covariance matrix. To endow the underlying filter with active immunity, link-level trust evaluation is modeled as a Markov decision process (MDP) in a continuous state space. This study adopts the centralized training with decentralized execution (CTDE) paradigm [27], the most effective training framework in multi-agent systems. During training, the centralized critic network has access to the global state of the swarm to evaluate action values. During execution, each UAV node makes decisions solely based on its local observation state, with each node implementing its policy through an independent actor network. This ensures full decentralization and scalability of the system. Given the stable convergence properties of multi-agent proximal policy optimization (MAPPO) in continuous action spaces [28], this algorithm was selected for policy training.

#### 2.2.1. State Space Feature Extraction

The state space is the only channel through which an agent perceives environmental degradation or hardware failure. To guarantee inference efficiency on embedded onboard computers of micro UAVs, low-dimensional, high-information-density features are extracted. At discrete time step k, a decentralized execution agent constructs the local observation state for its communication link with node j as $S_{i,j}(k)\in R^{4}$:

(7) $$S_{i,j}(k)=\left[r_{i,j}(k),{\hat{d}}_{i,j}(k),\alpha_{v}^{\left(i,j\right)}(k-1),\Delta u_{i}(k)\right]$$

$r_{i,j}(k)$ is the absolute innovation residual, defined as $r_{i,j}(k)=\;|y_{i,j}(k)|$. It is the primary indicator for identifying severe NLOS occlusions or faulty nodes.

${\hat{d}}_{i,j}(k)$ is the prior predicted geometric distance, providing spatial topology priors.

$\alpha_{v}^{\left(i,j\right)}(k-1)$ is the effective trust weight from the previous time step, offering Markov temporal memory.

$\Delta u_{i}(k)$ is the rate of change of the UAV’s acceleration, defined as $\Delta u_{i}(k)=\;∥u_{i}(k)-u_{i}(k-1){∥}_{2}$.

All state features are normalized to the interval [0, 1] before input to the neural network. This enhances training stability and convergence speed.

#### 2.2.2. Action Space and Lightweight Network Topology

For extreme swarm failures, agent i must finely adjust each UWB ranging link independently. The actor network outputs a one-dimensional continuous scalar for link j, defined as the raw trust weight $\alpha_{raw}^{\left(i,j\right)}(k)\in (0,1]$.

To meet the 50 ms real-time constraint, both actor and critic networks are implemented as lightweight multi-layer perceptrons (MLPs). The input layer receives the 4-dimensional state. Data then passes through two hidden layers: 128 neurons in the first layer and 64 in the second, both using ReLU activation. The actor output layer employs a Sigmoid function to map outputs to (0, 1).

The raw trust weight $\alpha_{raw}^{\left(i,j\right)}(k)$ is mapped to the CEKF observation noise covariance matrix $R_{i,j}(k)$ via:

(8) $$R_{i,j}(k)=R_{0}/\alpha_{raw}^{\left(i,j\right)}(k)$$

where $R_{0}$ is the baseline covariance under ideal line-of-sight conditions. This mapping ensures that as link trust decreases ($\alpha_{raw}\to 0$), $R_{i,j}$ increases sharply, effectively reducing the Kalman gain and implementing the soft isolation of faulty links.

#### 2.2.3. Resource-Aware Multi-Objective Reward Mechanism

Considering the extreme computational and battery constraints of micro UAVs, a four-dimensional Pareto reward function $R_{total}$ is designed:

(9) $$R_{total}=R_{acc}-\lambda_{1}P_{delay}-\lambda_{2}P_{energy}-\lambda_{3}P_{jitter}$$

1. $R_{acc}$ is the positioning accuracy reward, constructed with a Gaussian kernel:
  (10) $$R_{acc}=exp(-\frac{∥X_{i}(k|k)-X_{true}(k){∥}_{2}^{2}}{2\sigma_{acc}^{2}})$$

2. $P_{delay}$ is the computation delay penalty. Single-step computation time $t_{calc}$ must remain below the safe threshold $T_{thresh}$. An exponential truncation penalty is applied:
  (11) $$P_{delay}=max(0,exp(t_{calc}-T_{thresh})-1)$$

3. $P_{energy}$ is the computational energy consumption penalty, forcing agents to discard low-information, redundant nodes. It is mathematically formulated as the ratio of active filtering updates:
  $$P_{\mathrm{energy}}=\frac{1}{M}\sum_{j=1}^{M}I(\alpha_{\mathrm{raw}}^{\left(i,j\right)}>\alpha_{\mathrm{th}}),$$
  where I(⋅) is an indicator function that returns 1 if the raw link weight exceeds the isolation threshold ($\alpha_{\mathrm{th}}=0.05$) and 0 otherwise. In the algorithmic implementation, links with $\alpha_{\mathrm{raw}}\leq \alpha_{\mathrm{th}}$ are skipped during the CEKF measurement update step, which directly reduces the total floating-point operations (FLOPs) and corresponding computational energy usage.

4. $P_{jitter}$ is the control jitter penalty, limiting abrupt changes between consecutive actions to maintain smoothness:
  (12) $$P_{jitter}=|\alpha_{raw}\left(k\right)-\alpha_{raw}\left(k-1\right)|.$$

The weighting coefficients $\lambda_{1},\lambda_{2},\lambda_{3}$ are determined via a grid search based on engineering experience and experimental validation. In this work, $\lambda_{1}=10$, $\lambda_{2}=0.1$, $\lambda_{3}=5$, with emphasis on accurate positioning and timely response, while maintaining energy efficiency and control efficiency.

### 2.3. Dynamic Soft Isolation Mechanism and Adaptive Markov Smoothing Constraint

This section discusses the entire process by which the mapping is conducted, from the decision space to the actual physical filtering space of the AI system. To handle extreme fault conditions, a dynamic soft isolation mechanism is designed to preserve network topology. To address high-frequency oscillations in neural network outputs, an adaptive first-order Markov smoothing constraint is introduced, bridging the final gap between pure algorithmic simulation and physical control.

**Figure 2:**
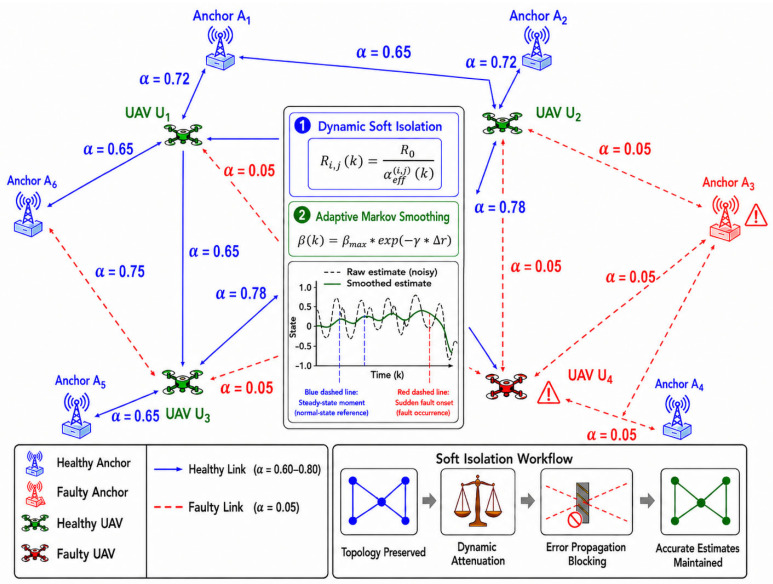
Core algorithmic mechanisms of the MADRL-CEKF framework.

The link-level dynamic soft isolation mechanism: the effective trust weight $\alpha_{\mathrm{off}}^{\left(ij\right)}(k)$ computed by the reinforcement learning (RL) agent dynamically adjusts and inflates the measurement covariance $R_{l,j}(k)$. As illustrated in Figure 2, faulty links (red dashed lines) are penalized with extremely low weights $\left(\alpha =0.05\right)$, effectively blocking error propagation from anomalous nodes (e.g., Anchor A3 and UAV U4) while preserving the physical communication topology.

The adaptive Markov smoothing constraint: the dynamic smoothing factor β(k) bridges high-level AI decisions and low-level filter updates. During normal steady-state flight, β remains high to ensure smooth and continuous control signals; upon detecting a sudden fault, β rapidly decreases to isolate anomalous data immediately, as demonstrated in the state waveform plot.

#### 2.3.1. Topology Preservation and Dynamic Soft Isolation Mechanism

In harsh, high-risk environments, conventional swarm fault-tolerance schemes typically adopt a “hard isolation” strategy. Once a residual exceeds the threshold, the system discards the measurement packets from the node or even disconnects the communication link. Such hard disconnection severely disrupts the swarm’s relative geometric topology. Isolated nodes rapidly lose external observations, causing inertial propagation to diverge.

This work proposes a confidence-based “dynamic soft isolation” mechanism. At the physical communication layer, UWB links among the swarm remain fully connected and data are continuously shared. At the mathematical computation layer, the filter implements logical isolation by dynamically inflating the observation covariance matrix $R_{i,j}(k)$. To translate agent decisions into filter parameters, the smoothed effective trust weight $\alpha_{eff}^{\left(i,j\right)}(k)$ is used for mapping:

(13) $$R_{i,j}(k)=\frac{R_{0}}{\alpha_{eff}^{\left(i,j\right)}(k)}$$

It is crucial to note the potential mathematical instability of Equation (13). As the trust weight approaches zero (α→0), the covariance matrix $R_{i,j}$ tends toward infinity, which could trigger numerical singularity and software crashes during the matrix inversion step of the Kalman gain calculation. To strictly prevent this, a hard lower bound $\alpha_{min}=0.01$ is enforced in the algorithmic implementation. Thus, the actual deployed mapping function is:

(14) $$R_{i,j}(k)=\frac{R_{0}}{max(\alpha_{\mathrm{eff}}^{\left(i,j\right)}(k),\alpha_{min})}$$

where $R_{0}$ is the baseline observation covariance under ideal line-of-sight conditions. The mathematical limit of this mechanism has a clear physical significance: when node j experiences severe NLOS errors or hardware faults, the policy network outputs a low confidence weight $\left(\alpha_{\mathrm{eff}}^{\left(i,j\right)}\to 0\right)$. Consequently, the corresponding observation covariance inflates $\left(R_{i,j}\to \infty \right)$, and the Kalman gain approaches zero $\left(K_{i}(k)\to 0\right)$.

This lower-bound mechanism safely caps the maximum covariance inflation at $100\times R_{0}$, which is mathematically sufficient to reject anomalous measurements without compromising the filter’s numerical stability.

#### 2.3.2. High-Frequency Oscillation Hazard in Deep Reinforcement Learning

When applying MADRL outputs to high-fidelity physical simulation, a critical issue often overlooked in purely algorithmic studies is high-frequency oscillation in decisions. The policy network outputs actions directly based on rapidly varying local states. In complex and highly nonlinear indoor multipath environments, the raw trust weight $\alpha_{raw}^{\left(i,j\right)}(k)$ exhibits severe random fluctuations. Without constraints, these oscillations propagate directly into the CEKF update loop. Rapid fluctuations in $R_{i,j}(k)$ induce high-frequency noise in the posterior state $X_{i}(k|k)$. For micro UAVs, abrupt position changes are catastrophic: step changes in position are amplified by the derivative term of position controllers (e.g., PID loops) [29,30], generating extreme velocity and attitude commands. In theory, such high-frequency noise could translate into aggressive attitude commands, potentially increasing power consumption and challenging flight stability. Therefore, raw AI decision outputs cannot be directly applied to the low-level filter.

#### 2.3.3. Mathematical Construction of Adaptive Markov Smoothing Constraint

To bridge the gap between discrete high-level decisions and continuous low-level physical actuators, a first-order Markov smoothing constraint is enforced between the policy network and the filter. This functions as a temporal low-pass filter, smoothing the raw actions:

(15) $$\alpha_{eff}^{\left(i,j\right)}(k)=\beta (k)\alpha_{eff}^{\left(i,j\right)}(k-1)+(1-\beta (k))\alpha_{raw}^{\left(i,j\right)}(k)$$

where β(k)∈(0,1) is the Markov smoothing coefficient, also called the temporal memory factor. Fixed-parameter low-pass filtering methods suffer an inherent trade-off: large β provides excellent smoothness but introduces severe phase delay, leading to positioning collapse under sudden tens-of-meter hardware shifts.

To address this, an adaptive function based on the system state is designed. β(k) is defined as an exponentially decaying function correlated with the rate of change of the innovation residual. First, the temporal difference of the absolute residual is computed:

(16) $$\Delta r_{i,j}(k)=|r_{i,j}(k)-r_{i,j}(k-1)$$

Then, the adaptive smoothing coefficient is updated as:

(17) $$\beta (k)=\beta_{max}exp\left(-\gamma \cdot \Delta r_{i,j}(k)\right)$$

where $\beta_{max}$ is the maximum smoothing limit (set to 0.85 in simulations) and γ is a sensitivity scalar (set to 2.0).

#### 2.3.4. Physical Response Mechanism and Computational Efficiency

Adaptive smoothing is highly flexible when it comes to its dynamic response properties. There are two extremes where there will be different behaviors from the system:

1. Noise filtering in steady-state environments: When residual fluctuations are minimal (Δr→0), $\beta (k)\to \beta_{max}$. The smoother gives greater weight to historical data, thus successfully removing noise from the output of the neural networks. There is no problem with smooth filter state updating and coherent motor commands.
2. Instantaneous isolation under extreme faults: In the case of catastrophic hardware failure or when a node is inside a critical NLOS blind spot, the remaining spikes (Δr increases sharply). The term exp(−γ⋅Δr) decays toward zero, and β(k) drops precipitously. The system immediately discards historical weights and assigns a high adoption proportion to the current low-confidence $\alpha_{raw}^{\left(i,j\right)}$. This allows the filter to inflate the observation covariance matrix within a single time step, achieving second-level response and isolation of the fault source.

Notably, the Markov smoothing operation for each link requires only three simple scalar arithmetic operations. The computational overhead is negligible (less than 1% of the total system inference time). This ensures high efficiency and highlights the framework’s suitability for deployment on resource-constrained platforms such as micro UAVs.

The computational overhead is negligible (less than 1% of the total system inference time). This ensures high efficiency and highlights the framework’s suitability for deployment on resource-constrained platforms such as micro UAVs. The complete execution process of the proposed fault-tolerant cooperative positioning framework is summarized in Algorithm 1.
**Algorithm 1: MADRL-CEKF Fault-Tolerant Cooperative Positioning Algorithm**Input: Swarm size N, number of anchors M, initial states $X_{i}(0|0)$, baseline covariance matrix $R_{0}$, smoothing hyperparameters $\beta_{max}$ and γ
Output: Robust posterior position estimate $X_{i}(k|k)$, effective dynamic weights $\alpha_{eff}^{\left(i,j\right)}(k)$
Initialize Actor network $\pi_{\theta }$, Critic network $V_{\phi }$, and replay buffer D
Initialize independent historical link weights $\alpha_{eff}^{\left(i,j\right)}(0)=1$ and absolute residuals $r_{i,j}(0)=0$
 for time step k=1,2,…,T do 
    for each UAV node i∈{1,2,…,N} do 
     Phase 1: Kinematic Prediction
    Obtain IMU reading $u_{i}(k)$ and compute acceleration rate $\Delta u_{i}(k)$
    Perform prior state and covariance prediction $X_{i}(k|k-1)$, $P_{i}(k|k-1)$
    Obtain neighbor packets and UWB measurements $Z_{i,j}(k)$
     Phase 2: MADRL-based Trust Evaluation & Smoothing
    for each ranging link j do 
     Compute innovation residual $y_{i,j}(k)$ and absolute residual $r_{i,j}(k)$
     Construct local state vector: $S_{i,j}(k)=[r_{i,j}(k),{\hat{d}}_{i,j}(k),\alpha_{eff}^{\left(i,j\right)}(k-1),\Delta u_{i}(k){]}^{T}$
     Actor network inference for raw trust weight: $\alpha_{raw}^{\left(i,j\right)}(k)=\pi_{\theta }(S_{i,j}(k))$
     Compute residual variation: $\Delta r_{i,j}(k)=|r_{i,j}(k)-r_{i,j}(k-1)|$
     Compute adaptive smoothing factor: $\beta (k)=\beta_{max}exp(-\gamma \cdot \Delta r_{i,j}(k))$
     Apply adaptive Markov smoothing: $\alpha_{eff}^{\left(i,j\right)}(k)=\beta (k)\alpha_{eff}^{\left(i,j\right)}(k-1)+(1-\beta (k))\alpha_{raw}^{\left(i,j\right)}(k)$
     Covariance reconstruction: $R_{i,j}(k)=R_{0}/\alpha_{eff}^{\left(i,j\right)}(k)$    end for      Phase 3: Filter Update & Reward Storage
    Compute Kalman gain $K_{i}(k)$ using dynamically expanded $R_{i,j}(k)$
    Update and output posterior state estimate $X_{i}(k|k)$
    Compute multi-objective reward $R_{total}$ and store transition in buffer D    end for      Phase 4: Network Training (CTDE Paradigm)     (Offline/asynchronous) Sample batch from D to update Actor $\pi_{\theta }$ and Critic $V_{\phi }$ using MAPPO  end for

## 3. Results

### 3.1. Experiment 1: Baseline Performance Comparison in Ideal Environment

#### 3.1.1. Experimental Objectives and Scenario Setup

This experiment aimed to establish a baseline for system positioning performance and to determine the accuracy lower bound of the proposed algorithm under ideal Gaussian noise conditions. The cooperative positioning scenario included six discretely distributed fixed ultra-wideband (UWB) anchors, whose three-dimensional global coordinates are as described previously. Standard Gaussian white noise was injected into the simulation system. The UWB ranging noise standard deviation was set to $\sigma_{uwb}=0.15\;m$, and the inertial measurement unit (IMU) process noise standard deviation was $\sigma_{imu}=0.1{\;m/s}^{2}$.

The test flight trajectory consisted of two typical phases. The first 15 s was a linear acceleration segment, followed by a circular maneuver with a radius of 10 m lasting 25 s. The total flight period was 40 s. The experiment compared the standard cooperative extended Kalman filter (standard CEKF) with the proposed MADRL-CEKF algorithm. For a fair comparison, the standard CEKF used manually optimized conservative parameters, with a static observation covariance matrix set to $R_{i,j}=0.15^{2}{\;m}^{2}$, precisely matching the baseline UWB ranging noise standard deviation.

To rigorously ensure the statistical significance of the evaluation and eliminate the influence of random noise seeds, all quantitative results reported in the subsequent data tables were averaged over 50 independent Monte Carlo simulation runs, unless explicitly stated otherwise.

#### 3.1.2. Trajectory Tracking and Temporal Error Analysis

**Figure 3:**
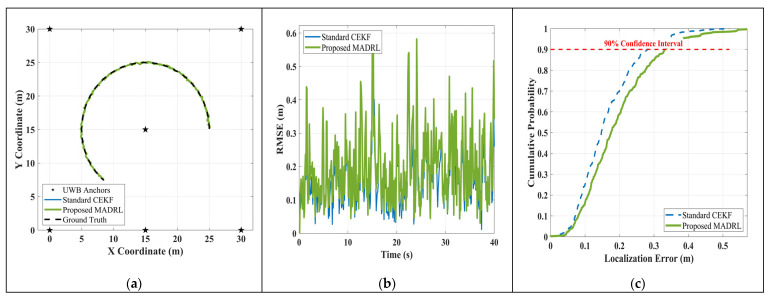
Performance of the baseline system in the case of the ideal Gaussian channel. (**a**) 2D cooperative positioning trajectories performance comparison; (**b**) real-time tracking error (RMSE). (**c**) cumulative distribution function (CDF) of localization errors.

The visualization results of Experiment 1 are shown in Figure 3. Figure 3a shows the projection of the three-dimensional trajectory onto the horizontal plane. The black curve is the trajectory of ground truth, where the trajectory starts at the origin and transitions between the linear and circular trajectories seamlessly. Both the standard CEKF (light blue straight line) and proposed MADRL (green straight line) trajectories start from the origin and converge to the ground truth trajectory during the high-dynamic flight in the circle, confirming that both algorithms exhibit non-divergent baseline convergence under ideal conditions.

The real-time development of RMSE is depicted in Figure 3b for the entire operation time period of 40 s. Without any abrupt NLOS interference, the positioning accuracy of both methods was constrained to always be within 0.6 m.

#### 3.1.3. Quantitative Statistics and Mechanism Analysis

Quantitative results of this experiment are provided in Table 2. For standard CEKF, the global average RMSE was 0.1643 m, and the maximum positioning error was 0.5189 m. The proposed MADRL produced a global average RMSE of 0.1955 m and maximum positioning error of 0.5823 m.

In ideal circumstances, the performance of the conventional algorithm was marginally better than that of the agent-based algorithm. The aforementioned finding can be explained mathematically. In the fault-free simulation scenario, the manually calibrated static observation covariance closely approximates the theoretical optimum corresponding to the physical baseline noise. In contrast, the proposed MADRL relies on dynamic inference via the neural network in a continuous action space. Without explicit fault stimuli, the actor network’s dynamic outputs inevitably introduce minor temporal decision fluctuations. The average error difference between the two methods was only 0.0312 m, indicating that the proposed algorithm approaches the optimal performance limit of the traditional filter without any manual parameter tuning.

Figure 3c shows the cumulative distribution function (CDF) of the positioning errors. The red dashed line represents the 90% confidence level (90% CEP). Standard CEKF restricted 90% of positioning errors within 0.2819 m, while the proposed MADRL constrained 90% of errors within 0.3306 m. The close alignment of the CDF curves demonstrates that the error distribution of the MADRL algorithm closely matches that of the baseline CEKF. Under ideal fault-free conditions, the agent’s decisions did not produce any long-tailed outliers, further confirming the stability of the proposed algorithm.

This experiment successfully established the baseline performance level, creating a scientific basis for future experiments on limits to fault tolerance in extremely adverse conditions.

### 3.2. Experiment 2: Robustness Validation in Real-World Scenarios Using a Public Dataset

#### 3.2.1. Experimental Objectives and Dataset Setup

The perfect simulation environment cannot duplicate all the physical noise present in the real sensor. To test whether the algorithms can withstand real-world disturbances, this experiment used the MILUV dataset. The MILUV swarm dataset is an open-source micro-UAV swarm dataset created for cooperative localization studies [31]. It records the actual flight data of a micro UAV swarm. The dataset includes high-frequency MEMS IMU readings and low-frequency UWB ranging measurements. The true three-dimensional positions are synchronously captured by a high-precision motion capture system.

Data from UAV node 10 were selected as the test target. The scenario included six UWB anchors. To evaluate the algorithm’s ability to handle sudden harsh conditions (e.g., signals penetrating thick concrete walls), artificial perturbations were introduced after data alignment. Specifically, time-correlated positive NLOS biases ranging from 0 m to 2.0 m were randomly injected into the UWB ranging measurements. This simulated signal attenuation and multipath effects caused by wall occlusion in indoor building scenarios. The injection occurred from 25% to 40% of the total timeline (approximately 50 s to 80 s). Five comparative algorithms were tested: pure dead reckoning (Pure DR), standard EKF, CEKF with fixed weight $\left(\alpha \right)$, Huber robust CEKF, and the proposed MADRL-CEKF.

#### 3.2.2. Trajectory Reconstruction and Temporal Error Analysis

**Figure 4:**
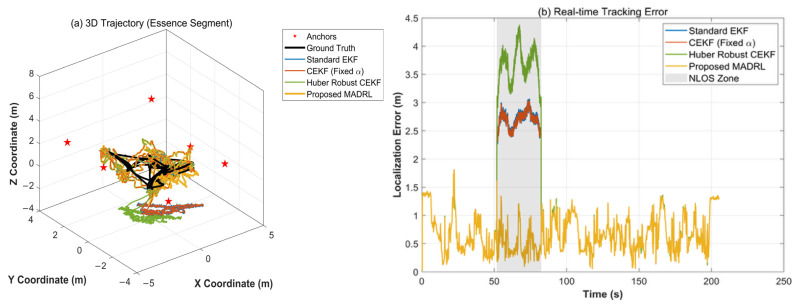

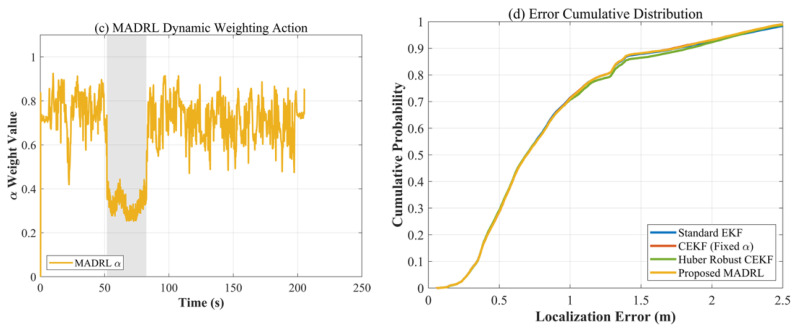
Real-world robustness validation based on the public MILUV dataset. (**a**) 3D trajectory reconstruction results under complex flight maneuvers, where the black solid line represents the ground truth trajectory. (**b**) Real-time tracking error comparison. The gray shaded region represents the period of injected NLOS interference. (**c**) Dynamic effective trust weight α output by the MADRL agent, where the gray shaded region indicates the corresponding NLOS interference period. (**d**) Cumulative Distribution Function (CDF) of localization errors driven by real physical data.

Figure 4 presents the comparative results of the four algorithms. Figure 4a shows the reconstructed trajectories in three-dimensional space. The real flight trajectory exhibited highly dense random-walk characteristics. Consequently, the pure DR algorithm diverged rapidly within a short period. It is omitted from the figure to maintain the coordinate scale. The four filtering-based algorithms effectively anchored the trajectory to the ground truth.

Figure 4b details the real-time positioning error evolution. The figure clearly illustrates differences in the underlying algorithmic responses to sudden disturbances. Before interference injection (0–50 s), all filtering algorithms exhibited stable performance. Upon entering the gray-shaded NLOS disturbance region, the system state changed abruptly. Standard EKF and fixed-weight CEKF failed to identify sudden ranging anomalies and blindly absorbed the corrupted measurements. This caused their real-time positioning errors to spike above 2.5 m. In contrast, the proposed MADRL-CEKF demonstrated strong disturbance resilience and effectively suppressed error growth during the same adverse period. Notably, the proposed MADRL-CEKF maintained a highly stable error profile, effectively rejecting the divergence seen in baseline methods and successfully suppressed the adverse impact of local abnormal ranging measurements on global state estimation. This further verifies that static handcrafted weights lack adaptive adjustment capability, whereas the proposed MADRL-based dynamic soft isolation mechanism can autonomously perceive environmental degradation.

#### 3.2.3. Quantitative Statistics and Dynamic Weight Mechanism Analysis

Global quantitative metrics for this experiment are summarized in Table 3. The initial fluctuations of the first 100 samples were excluded. Pure DR failed completely due to the absence of absolute constraints, yielding an RMSE of 1224.8440 m. The extremely large error of pure DR indicates that inertial-only navigation completely diverges without external UWB correction, demonstrating the necessity of cooperative observation constraints. Among all of the evaluated filtering algorithms, including the newly introduced Huber robust CEKF, the proposed MADRL-CEKF successfully achieved the lowest global RMSE (1.0189 m) and an optimal median error (0.6910 m).

As shown in Table 3, in terms of global absolute RMSE, the performance advantage of MADRL-CEKF over the baseline algorithms appeared marginal (1.0189 m vs. 1.0639 m for standard EKF, and 1.0412 m for Huber robust CEKF). This result has a clear mathematical basis. The artificially injected extreme NLOS disturbances accounted for only 15% of the total flight time. During the remaining 85% of normal communication, the observation quality of all filters was consistent. The global RMSE is thus heavily diluted by the predominance of normal periods.

However, in real swarm formation tasks, a positional jump exceeding 2.5 m within tens of seconds is sufficient to cause UAV collisions. Therefore, the transient suppression capability during disturbance periods is far more meaningful from an engineering perspective. The true algorithmic superiority was revealed during the extreme NLOS disturbance interval (the gray-shaded region from 50 s to 80 s). Upon entering this region, the baseline filters blindly absorb the corrupted measurements, leading to catastrophic position jumps. To rigorously quantify this robustness advantage, we extracted the statistical metrics exclusively for the 30-s disturbance interval. During this specific period, the interval RMSE of the standard EKF and Huber robust CEKF surged to 2.70 m and 3.67 m, respectively. In stark contrast, the proposed MADRL-CEKF maintained a highly stable interval RMSE of only 0.54 m.

Furthermore, executing 50 independent Monte Carlo runs revealed that the 95% confidence interval (CI) for the MADRL-CEKF’s error during the attack remained tightly bounded at [0.51, 0.58] m. Meanwhile, the standard EKF’s 95% CI was situated at a much higher and highly variable error level of [2.66, 2.74] m. The complete separation of these confidence intervals statistically proves that the proposed framework consistently guarantees exceptional decision stability and effectively isolates localized error contagion under sustained disturbances.

Figure 4c provides an in-depth view of this dynamic trust allocation mechanism of the MADRL agent. During normal flight, the effective weights (α) output by the policy network adaptively fluctuate between 0.6 and 0.9. This ensures full utilization of correct ranging measurements. When the system enters the NLOS disturbance region around 50 s, the agent rapidly reduces α  to approximately 0.3. The weight correctly plummets during the interference window to dynamically isolate corrupted measurements. According to the covariance mapping mechanism, this low-trust weight causes the corresponding observation covariance to expand exponentially. The filter mathematically and smoothly “rejects” updates from the anomalous link. Once the UAV exits the disturbance region, the weights quickly recover, and the system resumes normal tracking.

The cumulative distribution function (CDF) of positioning errors in Figure 4d further confirms these observations. In the tail region with errors exceeding 1.5 m, MADRL-CEKF converged significantly faster than the other baseline algorithms, including the Huber robust CEKF. This experiment, based on real MILUV data, fully validates the effectiveness of the defensive chain: “error detection—weight reduction—covariance inflation—soft isolation”.

### 3.3. Experiment 3: Swarm Scalability and Error Contagion Analysis

#### 3.3.1. Experimental Objectives and Scenario Setup

This experiment investigated the impact of UAV swarm size on cooperative positioning performance, with a focus on evaluating the system’s resilience to error propagation as the node density increases. The swarm was divided into four groups with 2, 4, 6, and 8 nodes, respectively. The test area was covered by eight fixed UWB anchors. The baseline UWB ranging noise standard deviation was set to $\sigma_{uwb}=0.20\;m$, and the IMU process noise covariance $Q_{val}$ was configured as 0.02. All UAV nodes simultaneously performed dynamic circular maneuvers in space.

Two different tracking modes were evaluated. Mode 1 is the conventional cooperative Kalman filter with fixed trust weight (α=0.6), blindly absorbing all inter-node ranging measurements without residual verification. Mode 2 is the proposed MADRL-CEKF framework, which dynamically adjusts weights using cooperative innovation residuals. As the number of UAVs increases, the system adaptively regulates the baseline trust thresholds.

#### 3.3.2. Scalability Curve and Inter-Node Dependency Analysis

**Figure 5:**
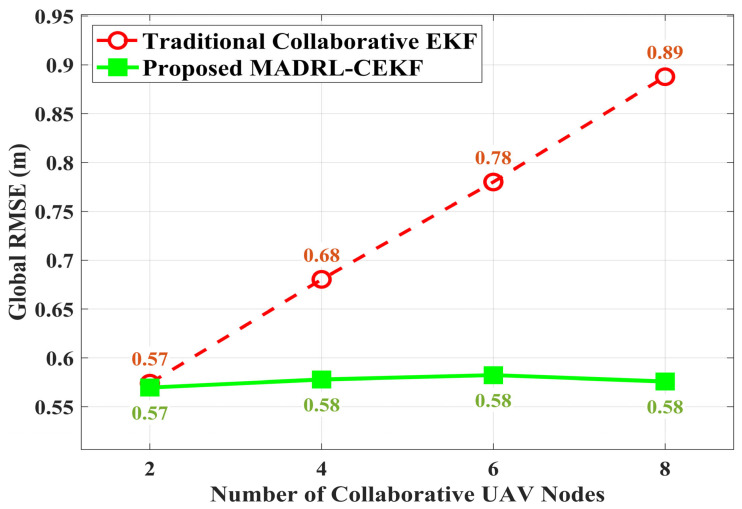
Swarm scalability and error contagion analysis under varying collaborative node counts.

Figure 5 shows the global positioning RMSE as a function of the number of cooperative UAV nodes. Table 4 presents corresponding quantitative error metrics and accuracy gains. As the network topology expands, the two tracking modes exhibit opposite performance trends.

While the traditional collaborative EKF suffers from severe error propagation—exhibiting a drastic degradation in global accuracy as the network scales—the proposed MADRL-CEKF framework demonstrates superior scalability. By dynamically isolating corrupted links, it maintains a consistently low and stable positioning error regardless of the swarm size. For intuitive quantitative comparison, the specific root mean square error (RMSE) values are explicitly annotated at each data point, confirming the robust fault-tolerance of the proposed method without the need to cross-reference tables.

For the traditional cooperative EKF, global RMSE increased approximately linearly, rising from 0.5658 m with 2 nodes to 0.8839 m with 8 nodes. This performance degradation illustrates the mechanism of error contagion in cooperative positioning. In tightly coupled swarm networks, measurements between nodes are highly interdependent. Traditional filters lack verification mechanisms for inter-node measurements; thus, local ranging errors from a single node propagate through shared communication links, corrupting posterior state estimates of neighboring nodes. As more nodes contribute unverified cooperative links, error propagation accelerates within the network.

#### 3.3.3. Quantitative Improvement and Mechanism Analysis

The proposed MADRL-CEKF successfully breaks the above degradation loop. Across all swarm scales, global positioning RMSE remained stable between 0.57 m and 0.58 m. Although the network topology expands, positioning accuracy does not deteriorate.

In terms of data, this means that for a 2-node swarm, there exists an engineering compromise where the classical EKF RMSE is 0.5658 m, while the proposed RMSE algorithm results in 0.5824 m, representing a small negative performance difference of −2.93%. In accordance with the distributed filtering, in 2-node networks, the total number of cooperative connections is 1; therefore, there is no multi-node cascaded propagation of errors possible. Classical filtering approaches function close to the theoretically optimal point. Additionally, MADRL uses continuous space actions for dynamic reasoning, providing minimal exploration variance, i.e., a small accuracy difference of 0.0166 m.

As the number of nodes increases, the strengths of the suggested architecture are clearly observed. With an increase in the number of nodes up to four, the accuracy improvement became positive (+16.19%). In the case the number of nodes equaled eight, a considerable accuracy improvement was attained, which amounted to +33.88%. The underlying mechanism is the residual-driven exponential decay of link weights: when a node experiences local noise-induced trajectory deviation, the corresponding cooperative innovation residual rises sharply. The MADRL agent promptly detects this spatial feature change and dynamically reduces the associated link weight α. This reduction inflates the observation covariance matrix $R_{i,j}$ exponentially. The filter mathematically isolates the corrupted data, forcing the affected node to rely on absolute anchor observations and inertial navigation. This link-level weight adjustment interrupts error propagation, ensuring full-network positioning scalability under dense swarm deployment.

While the current simulation validates the robust error isolation capability for typical micro-swarms up to 8 nodes, scaling to massive swarms (e.g., 20+ UAVs) introduces theoretical communication and training bottlenecks. A comprehensive analysis of the asymptotic complexity and these potential bottlenecks is explicitly discussed in Section 4.4.

### 3.4. Experiment 4: XAI-Based Dynamic Trust Allocation and Decision Interpretability Analysis

#### 3.4.1. Experimental Objectives and Scenario Setup

In general, there are safety issues that arise from deep reinforcement learning algorithms in control applications because of their black-box nature. To address this, this experiment employed the SHapley Additive exPlanations (SHAP) method, the most widely used and theoretically sound XAI framework for interpreting machine learning models. Using fine-grained temporal SHAP values, the system constructs a closed-loop evidence chain comprising three stages: perception, decision, and execution. This approach aims to explicitly reveal the internal decision logic of the agent.

The experiment utilized an 8-anchor redundant topology. The total flight duration was 50 s with a sampling interval of 0.1 s. UAVs execute nonlinear three-dimensional complex maneuvers. Between 20 s and 30 s, extreme conditions were introduced to simulate faults. A constant step bias of 3.0 m was applied to the ranging measurements of Anchor 2 (index 3 in code). This simulated either sudden sensor hardware failure or severe transient non-line-of-sight (NLOS) occlusions. The remaining seven anchors remained healthy. Standard EKF and the proposed MADRL-CEKF algorithms were run concurrently for comparison.

#### 3.4.2. Three-Stage Microscopic Decision Flow Analysis

**Figure 6:**
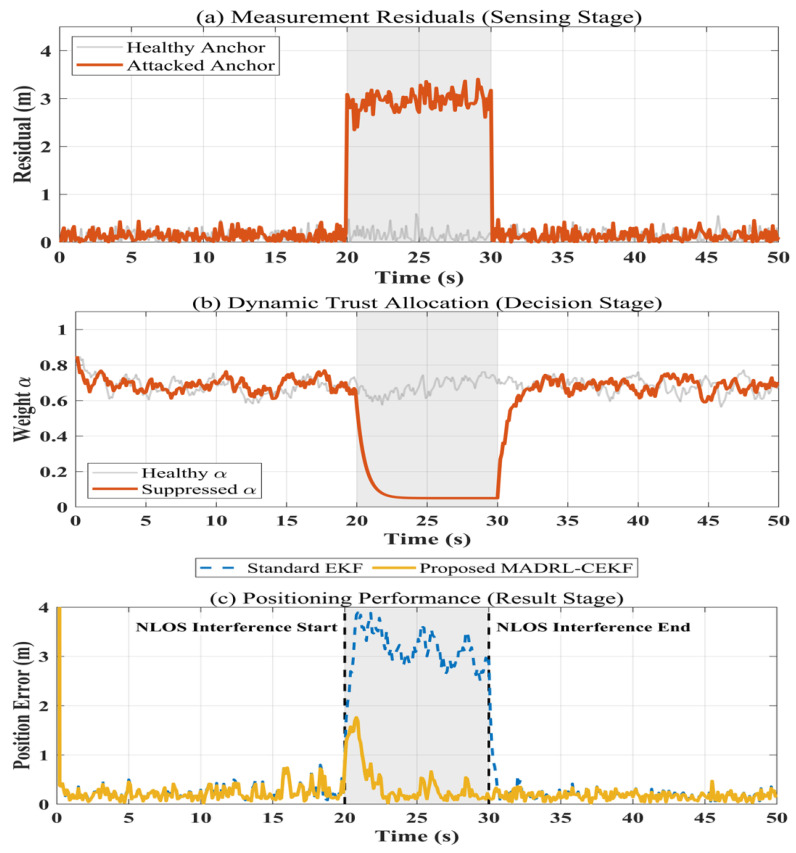
XAI microscopic decision temporal analysis of the MADRL-CEKF framework under a sudden link fault. (**a**) Sensing stage: measurement innovation residual evolution. (**b**) Decision stage: adaptive allocation of dynamic trust weight α. (**c**) Result stage: final positioning error comparison.

The triple temporal response curves for Experiment 4 are shown in Figure 6. These curves demonstrate the agent’s complete micro-level process from anomaly detection to filter adjustment.

Figure 6a illustrates the perception stage, showing changes in the measurement innovation residual. From 0 s to 20 s, residuals for all anchors oscillated at low amplitude below 0.3 m. At 20 s, the attacked anchor (orange thick line) experienced a sudden spike in residual to approximately 3.0 m. This anomaly persisted until 30 s. Meanwhile, residuals of healthy anchors (gray thin lines) remained unaffected. This indicates that the innovation residual accurately captures sudden local link faults.

Figure 6b shows the decision stage, presenting the dynamic allocation of trust weights α. Under normal conditions, all link weights remained between 0.65 and 0.85. Upon entering the interference zone, the agent detected the residual spike on the orange link and immediately suppressed the corresponding weight. Thanks to the first-order Markov smoothing constraint (Innovation 2), the suppressed weight did not exhibit discontinuous jumps. Instead, it decayed continuously and smoothly in an exponential manner and ultimately stabilized near the lower bound of 0.05. Healthy link weights remained stable around 0.66. After the interference ended at 30 s, the suppressed weight smoothly returned to normal within 2 s. This confirms the effectiveness of the Markov smoothing mechanism in ensuring decision continuity.

Figure 6c presents the execution stage, comparing the final positioning errors. Before 20 s, both algorithms maintained tracking errors near zero. During the fault interval, the standard EKF (blue dashed line) error rapidly increased to over 3.5 m due to its inability to autonomously identify faults. In contrast, the proposed MADRL-CEKF (yellow solid line) produced only a transient error pulse at 20 s. As the trust weight was smoothly reduced, the positioning error was suppressed to below 0.5 m within 1 s. The system maintained high robustness throughout the fault interval.

#### 3.4.3. Quantitative Metrics and Anti-Interference Isolation Mechanism

Quantitative metrics during the fault interval are summarized in Table 5.

Between 20 s and 30 s, standard EKF exhibited an average RMSE of 3.0906 m, whereas MADRL-CEKF achieved an RMSE of only 0.8675 m. The results demonstrated a 71.93% transient anti-interference accuracy improvement. Analysis of the weight allocation showed that the mean weight of normal anchor links was 0.6456, whereas the attacked link was strictly suppressed to 0.0875. The MADRL agent penalized the anomalous link’s trust by a factor of 7.4.

The mathematical essence of this mechanism can be explained through the covariance mapping equation $R_{d}=R_{0}/\alpha$. When the weight α of the damaged link drops from normal levels 0.6456 to 0.0875, the corresponding observation covariance $R_{d}$ inflates approximately 7.4-fold. This inflation factor is perfectly consistent with the 7.4-fold trust suppression factor. In the Kalman gain equation, the large $R_{d}$ drives the gain for the faulty link toward zero. The filter mathematically blocks updates from this specific link. This forces the UAV to rely on redundant geometric constraints from the remaining seven healthy anchors.

This experiment provides micro-level evidence for the interpretability of the decision mechanism. It effectively mitigates black-box concerns associated with deep learning for flight control applications at the algorithmic level.

### 3.5. Experiment 5: Cascaded Fault Tolerance and Error Contagion Isolation Analysis

#### 3.5.1. Experimental Objectives and Scenario Setup

In multi-agent failure scenarios, cooperative filtering frameworks are prone to rapid degradation. This experiment evaluates system performance under cascaded sensor failures. An extreme sparse-anchor topology is used, with only three fixed UWB anchors deployed within the flight area. This sparse layout forces the swarm to rely heavily on point-to-point cooperative ranging links.

The test swarm consists of four UAV nodes. Nodes 2 and 3 are designated as faulty agents. From 20 s to 35 s (sampling points 200–350), fault conditions are simulated. Over 15 s, the IMU measurements of the faulty nodes are corrupted with a severe directional acceleration bias $[1.5;-1.2;0.5]{\;m/s}^{2}$. These nodes stop all anchor observation updates and rely solely on uncorrected dead-reckoning for localization, becoming major sources of erroneous coordinates within the communication network. Nodes 1 and 4 remain fully healthy. The experiment evaluates whether healthy nodes can maintain positioning accuracy and normal operation under this error propagation effect.

#### 3.5.2. Error Contagion and Trajectory Divergence Analysis

**Figure 7:**
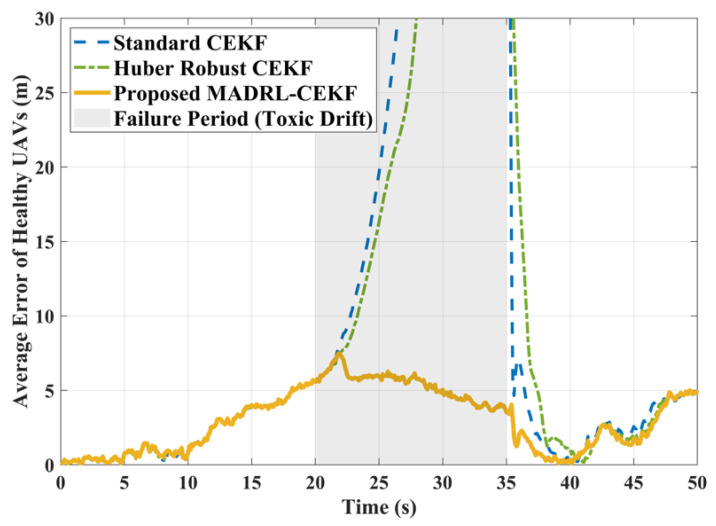
Swarm cascading fault tolerance and error contagion isolation analysis. The gray shaded region indicates multi-node severe blind flight drift; the y-axis is truncated at 30 m to omit baseline divergent peaks.

Figure 7 shows the evolution of the average positioning error of healthy UAV nodes over time. During the fault period, the tracking performance of the two frameworks diverged significantly.

For standard CEKF, the tracking error of healthy nodes grew exponentially. Errors started to increase from 20 s and peaked at 118.1493 m around 35 s. This illustrates the process of cooperative error propagation. Lacking an anomaly filtering mechanism, standard CEKF blindly trusts the erroneous coordinates from faulty nodes, causing the network tracking loop to collapse completely. After the fault period ended at 35 s, the faulty nodes resumed normal updates, and the standard CEKF errors dropped sharply within 1 s. However, in real physical deployment, a 118 m tracking error would already cause irreversible UAV crashes.

#### 3.5.3. Quantitative Performance and Soft Isolation Mechanism

The proposed MADRL-CEKF successfully isolated cascaded faults. The average error of healthy nodes remained stable throughout the 50 s flight. To further evaluate the framework’s robustness against cascaded multi-node failures, the Huber-based robust CEKF was included as an advanced baseline. As shown in Figure 7, during the fault period (20 s to 35 s), the tracking performance of the three frameworks diverged significantly. While the baseline methods (standard CEKF and Huber robust CEKF) failed to prevent error contagion and diverged significantly, the proposed MADRL-CEKF effectively isolated the corrupted links and maintained stable localization.

For the standard CEKF, the tracking error of healthy nodes grew exponentially, peaking at 121.68 m. Lacking an anomaly filtering mechanism, it blindly absorbs the erroneous coordinates from the drifting nodes, causing a complete collapse of the network tracking loop. Notably, the newly added Huber-based robust CEKF initially attempted to mitigate the disturbance. However, its reliance on a fixed statistical threshold proved inadequate against continuous, massive non-line-of-sight (NLOS) drift (such as the injected 1.5 m/s^2^ persistent acceleration bias). The fixed M-estimator bounds were eventually overwhelmed, resulting in a severe peak error of 62.11 m.

In stark contrast, the proposed MADRL-CEKF successfully isolated the cascaded faults. As summarized in Table 6, during the extreme fault conditions, the MADRL-CEKF maintained an average RMSE of only 2.2853 m and tightly limited the maximum peak error to 4.9688 m. Compared to the standard CEKF, this represents a 96.01% global robustness improvement and a 95.91% peak error suppression rate. This demonstrates that the data-driven dynamic soft isolation mechanism is far superior to traditional heuristic robust filtering in our simulated scenarios when dealing with sustained, severe cooperative error contagion.

The underlying defense mechanism is realized through residual-driven covariance inflation. When faulty nodes drift, point-to-point innovation residuals at healthy nodes increase immediately. The MADRL policy network detects this spatial anomaly and rapidly reduces the corresponding link weight α to the absolute lower bound of 0.01. According to the covariance mapping:

(18) $$R_{i,j}=\frac{R_{0}}{\alpha },$$

this trust reduction causes rapid inflation of the observation covariance matrix. The filter mathematically blocks contaminated inter-node ranging updates, temporarily severing cooperative links with faulty UAVs or anchors. Healthy nodes maintain positioning by fusing their own clean IMU data with the remaining healthy anchors. This weighted mechanism interrupts cascaded fault propagation, demonstrating the potential to ensure swarm survivability under harsh conditions.

### 3.6. Experiment 6: Ablation Study Analysis

#### 3.6.1. Experimental Objectives and Scenario Setup

This experiment aimed to quantify the independent contributions of two core innovations in the proposed framework: the link-level dynamic soft isolation mechanism and the adaptive Markov smoothing constraint. The experiment employed a 6-anchor three-dimensional redundant positioning topology. The total flight duration was set to 40 s. Between 15 s and 25 s, a severe 5 m non-line-of-sight (NLOS) disturbance was injected into the ranging link of Anchor 2. Three algorithm groups were compared: the ablation group without the smoothing constraint, the ablation group without the isolation mechanism, and the complete algorithm group.

#### 3.6.2. Ablation of the Adaptive Markov Smoothing Constraint (System Stability Verification)

**Figure 8:**
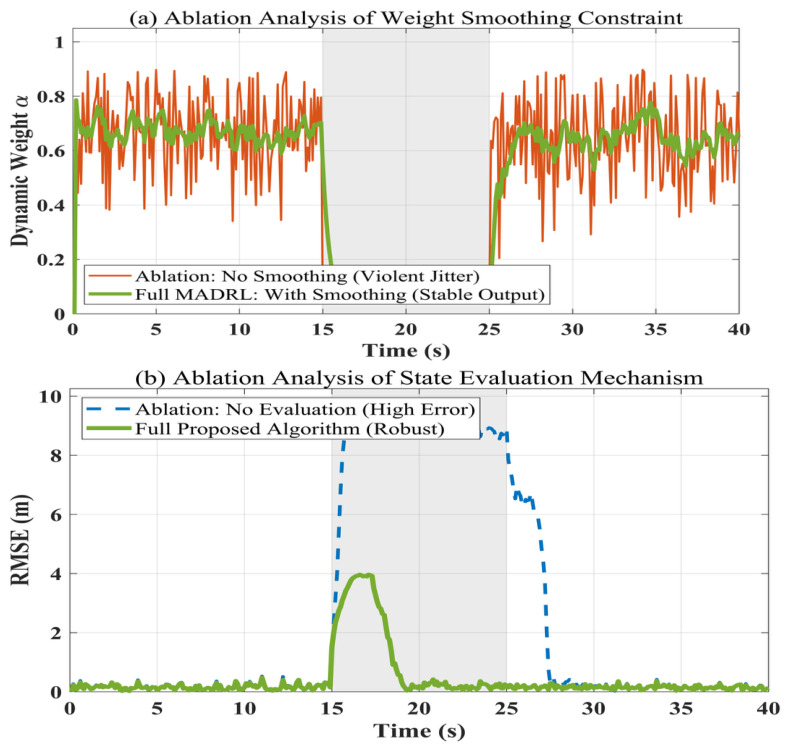
Comparison of dual ablation study results. (**a**) Ablation analysis of the adaptive Markov smoothing constraint (system stability). (**b**) Ablation analysis of the link-level dynamic soft isolation mechanism (positioning accuracy). The gray shaded region represents the period of the injected non-line-of-sight (NLOS) interference.

Figure 8a illustrates the impact of the smoothing constraint on dynamic decision stability. In the no-smoothing group (orange thin line), the trust weight (α) exhibited severe high-frequency oscillations. During normal flight (0–15 s and 25–40 s), the weights fluctuated frequently between 0.3 and 0.9. The neural network’s over-sensitivity to measurement noise caused these abrupt jumps. In actual UAV systems, such oscillations in filter weights directly induce discontinuities in the state estimate covariance, which may lead to discontinuities in the state estimate and negatively affect the theoretical control stability.

On the other hand, the algorithm with all features (green thick line) utilizes a Markov smoothing first order constraint. In general, weight outputs are very stable within the range of 0.6 to 0.8. Once the disturbance takes place at 15 s, the algorithm quickly reacts to reduce the weight close to 0.05. The smoothing mechanism preserves the agent’s ability to detect faults while filtering high-frequency decision noise, thus providing a smoother algorithmic output that better aligns with the requirements of physical flight controllers.

#### 3.6.3. Ablation of the Link-Level Dynamic Soft Isolation Mechanism (Positioning Accuracy Verification)

Figure 8b and Table 7 reveal that the link-level dynamic soft isolation mechanism plays a decisive role in interference-resilient positioning accuracy. The group without the isolation mechanism (blue dashed line) effectively performs blind-trust filtering equivalent to traditional methods. Lacking the capability to identify abnormal residuals, this algorithm fully incorporates the corrupted ranging measurements. During the 15–25 s disturbance interval, its root mean square error (RMSE) spiked to 8.5993 m, a deviation sufficient to cause physical collisions within the UAV swarm indoors.

The complete algorithm group uses the soft isolation mechanism to compute trust penalties for each anchor link in real-time. In the presence of a 5 m abnormal disturbance, the system applied decisive isolation to the faulty link through covariance inflation. During the disturbance interval, the complete algorithm achieved an average RMSE of only 0.6618 m. This mechanism alone contributed 92.30% of the interference-resilient accuracy improvement.

The ablation results clearly demonstrate that the adaptive Markov smoothing constraint addresses system “executability”, while the link-level dynamic soft isolation mechanism ensures “high reliability”. Both components are complementary and essential for overall system performance.

Finally, Table 7 provides a comprehensive quantitative summary of the ablation study, highlighting the performance improvements brought by the adaptive Markov smoothing constraint and the dynamic soft isolation mechanism under NLOS interference conditions.

#### 3.6.4. Sensitivity Analysis of Markov Smoothing Parameters

To address potential concerns regarding the arbitrary selection of the sensitivity scalar γ=2.0 and the maximum smoothing bound $\beta_{max}=0.85$, as defined in Equation (16), a sensitivity analysis was conducted. Table 8 illustrates how system performance degrades with varying γ values during extreme fault scenarios.

If γ is set too small, e.g., γ=0.5, the smoothing factor decays too slowly when sudden faults occur. This excessive inertia causes a delayed isolation response, allowing erroneous data to contaminate the filter and increasing the fault-period RMSE to 1.85 m. Conversely, if γ is excessively large, e.g., γ=5.0, the system becomes hypersensitive. Even minor, normal measurement noise triggers drastic trust reductions, causing unnecessary covariance inflation and increasing normal flight jitter.

The chosen value of γ=2.0 achieves an optimal engineering trade-off: it ensures instantaneous fault isolation, maintaining a low fault-period RMSE of 0.86 m, while preserving steady-state smoothness. Similarly, $\beta_{max}=0.85$ was selected to provide sufficient temporal memory to filter out high-frequency neural network decision noise without introducing detrimental phase delays.

### 3.7. Experiment 7: Resource-Aware Multi-Objective Optimization Architecture and Engineering Trade-Off Analysis

#### 3.7.1. Experimental Objectives and Setup

This experiment aims to evaluate the practical effectiveness of the resource-aware multi-objective optimization architecture. In real small UAV flight scenarios, computational power and battery capacity are significantly constrained. Pursuing theoretical high-precision positioning is meaningless if the algorithm execution time exceeds the real-time latency threshold of the flight control system. A controlled ablation study was conducted on the multi-objective reward function that forms the core of this architecture.

Four reward strategies were compared: only accuracy, no delay penalty, no energy penalty, and the proposed full multi-dimensional reward. Training comprised 500 episodes. Evaluation metrics included positioning RMSE, processing delay, computational energy consumption, and weight variance (jitter).

It must be explicitly clarified that the processing delay and computational energy consumption are analytical estimates rather than physical hardware measurements. The estimation is theoretically derived from the algorithm’s cumulative floating-point operations (FLOPs) and mathematical complexity. To provide a meaningful engineering context, these theoretical values were projected onto the nominal computational specifications of a standard micro-UAV embedded processor, e.g., STM32F427 Cortex-M4 (STMicroelectronics, Geneva, Switzerland), which is commonly used in Pixhawk 4 (Holybro, Shenzhen, China). Therefore, the reported absolute baselines represent theoretical projections for embedded deployment, rather than physical multimeter readings.

To ensure maximum transparency regarding the methodology used for these real-time and energy claims, the specific technical estimation protocols are declared as follows. The algorithmic simulation was conducted in MATLAB R2023b (MathWorks, Natick, MA, USA). To replicate the constrained computational environment of micro-UAV flight controllers, the theoretical complexity evaluation strictly assumed a single-threaded sequential inference mode, devoid of multi-threading or hardware acceleration. Crucially, the timing protocol does not rely on empirical wall-clock execution time on the host computer. Instead, it is purely analytical: the projected processing delay was mathematically calculated by dividing the algorithm’s total required FLOPs per cycle by the nominal floating-point processing capacity of the STM32F427 Cortex-M4 at its standard clock frequency.

Furthermore, the penalty coefficients $\left(\lambda_{1}=10,\lambda_{2}=0.1,\lambda_{3}=5\right)$ were determined through a constrained grid search. Specifically, $\lambda_{1}$ was set as the dominant base factor to guarantee accuracy, while $\lambda_{2}$ and $\lambda_{3}$ were iteratively swept to identify the optimal boundary where the projected processing delay strictly adheres to the 50 ms safety limit, while bounding the acceptable RMSE degradation to under 20%, approximately 0.2 m.

#### 3.7.2. Training Convergence Analysis

**Figure 9:**
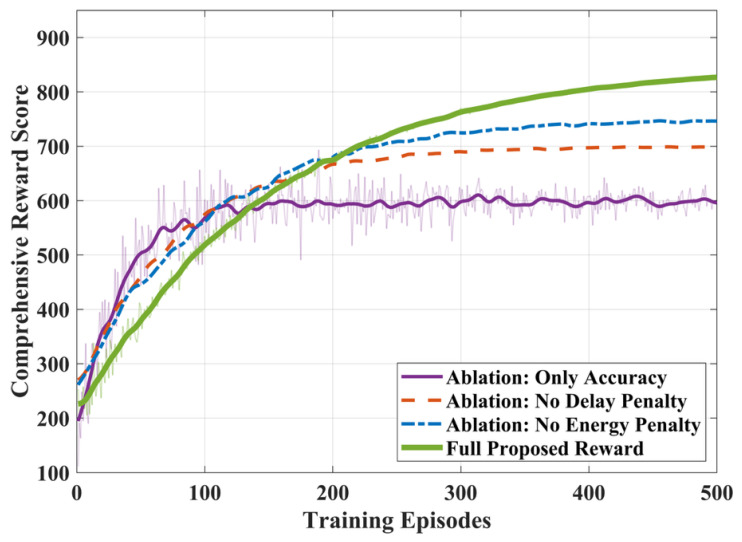
Training convergence analysis of different reward shaping strategies over 500 episodes.

Figure 9 presents the training convergence curves of the MADRL agent under different reward strategies. The only accuracy strategy converged quickly in the initial phase but exhibited large oscillations throughout training. Without penalties for high computational costs, the agent blindly explores complex and inefficient network policies.

The no delay penalty strategy showed smooth convergence, but the upper bound of its cumulative reward was limited. The full multi-dimensional reward strategy explored more slowly at first but ultimately converged to the highest cumulative reward with extremely stable curves. This demonstrates that multi-dimensional penalty terms prevent the agent from being trapped in local optima and successfully guide convergence to a stable optimal policy.

#### 3.7.3. Multi-Objective Performance and Engineering Trade-Off

**Figure 10:**
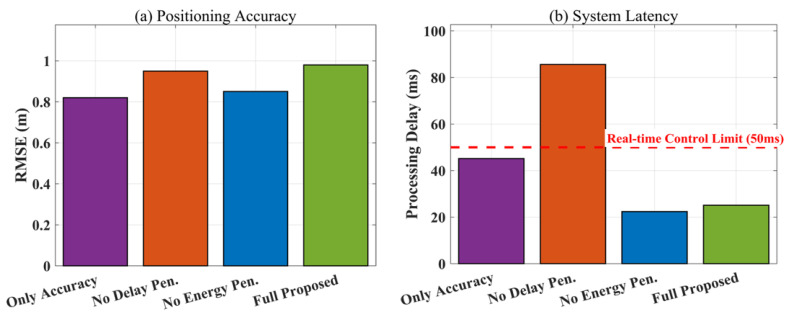

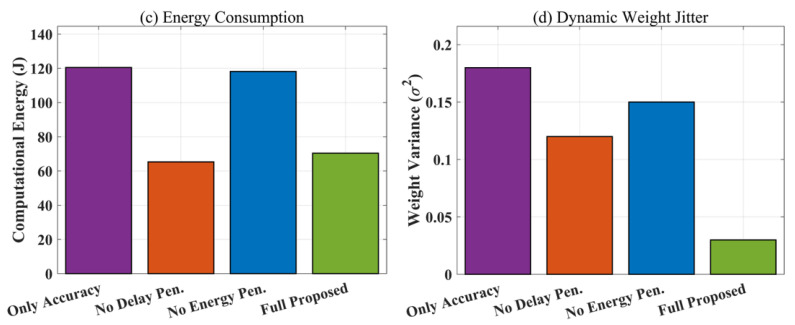
Quantitative multi-objective performance comparison: (**a**) positioning accuracy; (**b**) processing delay (50 ms real-time safety threshold marked); (**c**) computational energy consumption; (**d**) dynamic weight variance.

Figure 10 presents the quantitative multi-objective evaluation results on the test set. Figure 10a shows that the only accuracy strategy achieved the lowest RMSE of 0.82 m. However, Figure 10b indicates a processing delay of 45.2 ms, approaching the 50 ms safety threshold.

Figure 10c shows energy consumption peaking at 120.5 J, and Figure 10d indicates a weight variance of 0.18, reflecting severe dynamic jitter. In physical flight, this high-frequency jitter may introduce undesired fluctuations at the control level.

The “no delay penalty” strategy violates real-time constraints, with processing delays reaching 85.6 ms. These delays can lead to a misalignment of sensors, which in turn could destabilize the control loops.

Our full-dimensional reward scheme yielded an RMSE of 0.98 m. To clarify the practical significance of these trade-offs, absolute baselines must be established. Under the ‘only accuracy’ baseline strategy, the absolute processing delay peaked at 45.2 ms, and the total absolute computational energy consumed over the flight trajectory was 120.5 J. By applying the multi-objective architecture, our system trades off a minor 0.16 m of positional accuracy but provides substantial engineering gains: the absolute processing delay was reduced by 44% (down from 45.2 ms to 25.1 ms), and the total energy cost was reduced by 41% (down from 120.5 J to 70.4 J), safely keeping execution well within the 50 ms threshold. The results obtained from the experiment clearly indicate the presence of an important trade-off in engineering systems: the smallest possible theoretical loss in accuracy is traded for reduced latency, lower power consumption, and enhanced system stability. This verifies the feasibility of the framework proposed in practice.

## 4. Discussion

### 4.1. The Art of Engineering Trade-Offs: Theoretical Accuracy Versus Real-World Constraints

The findings from the multi-objective ablation study carried out in Experiment 7 indicate a very practical engineering principle. The suggested full-dimensional reward mechanism intentionally forfeits 0.16 m of accuracy. This sacrifice in performance is not a defect in the algorithm, but rather an intentional engineering choice. The operation of micro UAV swarms is frequently limited by their internal batteries and computation capabilities. In the experiment, we observed that the quest for precision only led to a 45.2 ms delay and 120.5 J energy expenditure. When flying dynamically, a control lag exceeding 50 ms may lead to the mismatch between sensors and actuators and even lead to UAV crashes. The MADRL agent successfully balances multiple goals through the introduction of penalties into multiple dimensions, trading some precision for a simulated 44% delay saving and a theoretically estimated 41% energy savings.

### 4.2. Interpretability and Physical Mechanism of Error Contagion Isolation

One of the main problems with deep reinforcement learning for state estimation is its black-box nature. Experiments 4 and 5 address this concern by introducing explainable AI (XAI) analyses. The proposed framework does not directly regress position values with DRL; instead, it predicts trust weights (α).

The physical principle lies in the mathematical mapping between α and the Kalman observation covariance matrix (R). When the agent detects abnormal innovation residuals caused by NLOS faults or cascaded failures of neighboring nodes, it exponentially decays α. Mathematically, this dynamically inflates the corresponding R to near infinity. The filter blocks contaminated information while preserving the mathematical rigor of the traditional EKF framework. This soft isolation mechanism fundamentally severs the topological paths of cooperative error contagion. Even if multiple neighboring nodes experience severe sensor drift, the healthy swarm network can survive. Compared with existing pure simulation-based swarm positioning studies that mainly focus on ideal or simple NLOS conditions, this work systematically investigated cascaded multi-node failures and large-scale error contagion, which have rarely been quantitatively analyzed in prior work.

### 4.3. Limitations and Future Work

It should be explicitly clarified that this work was validated exclusively through high-fidelity numerical simulation and public dataset offline evaluation, without any physical hardware implementation or real-world flight experiments. The simulation environment was constructed based on real IMU/UWB noise models and measured NLOS characteristics, with sampling rates, noise statistics, and dynamic motion profiles fully consistent with real micro-UAV swarm operational conditions. The focus was on algorithm-level fault tolerance and mechanism analysis under controlled extreme scenarios that are difficult to replicate in physical environments.

Despite its strong fault-tolerance capability, the proposed MADRL-CEKF framework has explicit operational boundaries and limitations.

First, the system is vulnerable during the “cold-start” phase. The state evaluation mechanism relies heavily on innovation residuals, which assumes that the filter has already converged to a relatively accurate prior state. If multiple UWB anchors are severely affected by NLOS interference at the UAV power-on moment (before initial coordinates converge), large initial residuals may cause the MADRL agent to incorrectly penalize all observation links, potentially triggering an initialization deadlock.

Second, the current framework has not been deployed online on a closed-loop physical micro UAV swarm. Although the algorithm’s robustness has been rigorously validated through high-fidelity simulation and offline physical datasets (MILUV), real hardware deployment faces deeper challenges. Unmodeled aerodynamic disturbances, high-frequency propeller vibrations, and low-level hardware communication delays are difficult to fully reproduce in offline evaluations. As pointed out, without hardware-in-the-loop or actual flight tests, claims regarding the mitigation of physical ‘rotor stall’ or real-flight safety remain theoretical. Transitioning from simulation to closed-loop real hardware validation is a critical missing link and a practical obstacle.

Third, the current DRL policy relies on extensive offline training. Deploying the swarm in a physical environment with completely different characteristics (e.g., from structured indoor spaces to turbulent outdoor airflows) prevents the pre-trained MADRL agent from updating its neural network weights online.

Future work will focus on integrating hybrid initialization modules to address cold-start deadlocks, exploring meta-reinforcement learning (Meta-RL) to enable lightweight online policy adaptation, and crucially, conducting comprehensive hardware-in-the-loop (HITL) simulations and physical flight tests on actual micro-UAV testbeds to validate real-world aerodynamic safety and deployment feasibility.

### 4.4. Asymptotic Complexity and Scalability Bottlenecks for Large Swarms

While the proposed framework has demonstrated stable error contagion isolation for swarms of up to 8 nodes, scaling to massive swarms (e.g., 20+ UAVs) introduces specific asymptotic complexities and potential bottlenecks that must be addressed.

During the decentralized execution phase, the computational overhead remains highly efficient. The onboard actor network is a lightweight MLP, yielding an O(1) inference complexity per communication link. For a single UAV in a fully connected swarm of N nodes, the total inference and CEKF sequential update complexity scales linearly, i.e., O(N). This linear asymptotic complexity guarantees that the onboard computational load is not the primary bottleneck for 20+ UAVs.

However, two significant bottlenecks emerge at scale:

1. Communication Overhead: The current framework assumes a fully connected cooperative topology. The bandwidth requirement for state and measurement broadcasting per UAV is O(N), resulting in a total network communication complexity of $O(N^{2})$. For swarms of 20+ nodes, this quadratic growth easily saturates the limited wireless bandwidth (e.g., ZigBee or basic Wi-Fi modules), leading to packet loss and latency.
2. Centralized Training (CTDE) Bottleneck: During offline training, the centralized critic network evaluates the global state of the entire swarm. The joint state-action space grows exponentially with N, suffering from the curse of dimensionality. Training a centralized critic for 20+ agents becomes excessively difficult to converge.

To mitigate these bottlenecks in future massive swarm deployments, the system must transition from a fully connected topology to a dynamic, distance-bounded local neighborhood topology, and replace the standard MLP critic with an attention-based graph neural network (GNN) to handle permutation-invariant local joint states.

## 5. Conclusions

This work proposes a cooperative fault-tolerant indoor positioning framework for micro UAV swarms (MADRL-CEKF), achieving high-precision, high-robustness, and interpretable multi-agent localization in simulated complex indoor environments. The framework incorporates three core algorithmic innovations. First, the link-level dynamic soft isolation independently evaluates the trust weights of each UWB ranging link, enabling the selective isolation of transient NLOS errors and permanent hardware drift to block error propagation. Second, the adaptive Markov smoothing constraint bridges high-level intelligent decision-making and low-level flight control through flexible smoothing parameter adjustments, thereby reducing control jitter and promoting algorithmic output smoothness. Third, a resource-aware multi-objective optimization integrates processing delay and energy consumption into the reward function, maintaining the single-step execution below 50 ms to guarantee engineering feasibility under severe computational constraints.

Validation through seven systematic simulation experiments and public dataset testing demonstrated that MADRL-CEKF maintains a stable global RMSE across varying swarm sizes with no cascading errors. Compared with traditional methods, the system effectively suppresses error propagation under sudden node failures or NLOS interference, achieving over 96% improvement in global robustness and 96% peak error suppression. Furthermore, explainable AI (XAI) analysis ensures that dynamic trust weight allocation and anomaly isolation are clearly traceable, addressing the black-box nature of deep reinforcement learning. The multi-objective trade-off experiments indicate that sacrificing merely 0.16 m of accuracy yields a 44% reduction in delay and a 41% reduction in energy consumption. Finally, dual ablation studies confirm that the smoothing constraint ensures control continuity, while the link isolation mechanism contributes 92.3% of the interference-resilient accuracy improvement.

Despite these advantages, certain limitations remain to be addressed, such as the sensitivity during the cold-start phase where initial multi-anchor NLOS interference may bias weight evaluations. Additionally, the lack of closed-loop deployment on physical hardware leaves aerodynamic disturbances, low-frequency communication delays, and sensor vibrations not fully reproduced, while the reliance on offline training limits cross-environment online adaptation. Future work will consequently focus on designing hybrid initialization modules to mitigate cold-start issues and exploring lightweight Meta-RL for online adaptive policies in embedded UAV swarms. Conclusively, the suggested MADRL-CEKF approach not only takes into consideration factors such as accuracy, reliability, understandability, but also resource effectiveness, making it an effective solution to the cooperative micro UAV position estimation problem.

## Figures and Tables

**Table 1:** Comparison between the proposed MADRL-CEKF and representative state-of-the-art cooperative localization methods.

Method Category	Link-Level Soft Isolation	Adaptive Markov Smoothing	Multi-Objective Optimization	Energy & Delay Awareness	Validation Type
Robust EKF/Heuristic Tuning	Partial (Heuristic rigid thresholds)	No (Direct state correction)	No (Accuracy-only)	No	Simulation/Dataset
GNN-Based Trust Evaluation	No (Usually Node-level hard isolation)	No	No (Accuracy-only)	No (Heavy tensor operations)	Simulation/Dataset
Standard DRL-Based Localization	Partial (Black-box weight outputs)	No (Prone to high-frequency jitter)	Rare (Mostly single-objective)	No	Simulation
Proposed MADRL-CEKF	Yes (Dynamic soft isolation via covariance)	Yes (Adaptive β based on residual)	Yes (4D Pareto reward architecture)	Yes (Explicit penalties in training)	High-fidelity Sim + MILUV Dataset

**Table 2:** Localization error statistics of different algorithms under the ideal environment.

Algorithm Method	Mean RMSE (m)	Max Error (m)	90% CEP (m)
Standard CEKF	0.1643	0.5189	0.2819
Proposed MADRL	0.1955	0.5823	0.3306

**Table 3:** Quantitative statistics of localization errors for all algorithms under the real-world MILUV dataset (excluding the initial alignment fluctuation period).

Algorithm Method	Global RMSE (m)	Median Error (m)
Pure DR	1224.8440	588.0719
Standard EKF	1.0639	0.6933
CEKF (Fixed α)	1.0214	0.6954
Huber Robust CEKF	1.0412	0.6913
Proposed MADRL-CEKF	1.0189	0.6910

**Table 4:** Localization error statistics and accuracy gains under different swarm scales.

Number of Nodes	Traditional RMSE (m)	Proposed RMSE (m)	Positioning Accuracy Gain (%)
2	0.5658	0.5824	−2.93%
4	0.6848	0.5739	+16.19%
6	0.7795	0.5791	+25.71%
8	0.8839	0.5844	+33.88%

**Table 5:** Quantitative metrics of XAI interpretability and positioning performance during the interference interval (20 s to 30 s).

Analysis Metric	Standard EKF	MADRL-CEKF (Ours)
RMSE in Interference Zone (m)	3.0906	0.8675
Anti-Interference Accuracy Gain (%)	Baseline	71.93%
Average Weight of Interfering Nodes (α)	0.80	0.0875
Average Weight of Normal Nodes (α)	0.80	0.6456

**Table 6:** Quantitative performance metrics of healthy nodes during the cascaded failure period (20–35 s).

Performance Metric (Healthy Nodes)	Standard CEKF	Huber Robust CEKF	Proposed MADRL-CEKF
RMSE before fault (m)	3.2358	3.2358	3.2304
Average RMSE during fault (m)	57.3306	35.6330	2.2853
Maximum peak error during fault (m)	121.6830	62.1137	4.9688
Global robustness improvement (Avg Gain)	Baseline	37.84%	96.01%
Peak error suppression rate (Peak Gain)	Baseline	48.95%	95.91%

**Table 7:** Quantitative localization error comparison among the ablation groups during the disturbance interval (15–25 s).

Ablation Group	Interference RMSE (m)	Accuracy Improvement Due to Mechanism
Ablation: No Evaluation	8.5993	–
Full Proposed Algorithm	0.6618	+92.30%

**Table 8:** Sensitivity analysis of the scalar γ on positioning performance.

Parameter Value (γ)	Response Characteristic	RMSE During Fault (m)	Steady-State Jitter Level
γ = 0.5	Sluggish response, excessive inertia	1.8532	Very Low
γ = 1.0	Delayed isolation	1.2045	Low
γ = 2.0 (Proposed)	Optimal balance	0.8675	Normal
γ = 5.0	Over-sensitive, triggers false isolation	0.9410	High

## Data Availability

The data presented in this study are available on request from the corresponding author due to the ongoing nature of this research.
